# Infective endocarditis: Do we have an effective risk score model? A systematic review

**DOI:** 10.3389/fcvm.2023.1093363

**Published:** 2023-02-20

**Authors:** Victoria Rizzo, Mohammad Yousuf Salmasi, Michael Sabetai, Christopher Primus, Jonathan Sandoe, Michael Lewis, Simon Woldman, Thanos Athanasiou

**Affiliations:** ^1^Cardiothoracic Surgery, St. Thomas Hospital, Guy’s and St Thomas’ NHS Foundation Trust, London, United Kingdom; ^2^Department of Cardiothoracic Surgery, Hammersmith Hospital, Imperial College Healthcare NHS Trust, London, United Kingdom; ^3^Specialised Cardiology, St Bartholomew’s Hospital, Barts Health NHS Trust, London, United Kingdom; ^4^Department of Microbiology, Leeds Teaching Hospitals NHS Trust, Leeds, United Kingdom; ^5^Department of Cardiothoracic Surgery, Royal Sussex County Hospital, Brighton and Sussex University Hospitals NHS Trust, Brighton, United Kingdom

**Keywords:** endocarditis, outcome assessment, risk score, risk factors, cardiac surgery

## Abstract

**Background:**

Infective endocarditis (IE) is a rare, highly morbid condition with 17% in-hospital mortality. A total of 25–30% require surgery and there is ongoing debate with regard to markers predicting patient outcomes and guiding intervention. This systematic review aims to evaluate all IE risk scores currently available.

**Methods:**

Standard methodology (PRISMA guideline) was used. Papers with risk score analysis for IE patients were included, with attention to studies reporting area under the receiver-operating characteristic curve (AUC/ROC). Qualitative analysis was carried out, including assessment of validation processes and comparison of these results to original derivation cohorts where available. Risk-of-bias analysis illustrated according to PROBAST guidelines.

**Results:**

Of 75 articles initially identified, 32 papers were analyzed for a total of 20 proposed scores (range 66–13,000 patients), 14 of which were specific for IE. The number of variables per score ranged from 3 to 14 with only 50% including microbiological variables and 15% including biomarkers. The following scores had good performance (AUC > 0.8) in studies proposing the score (often the derivation cohort); however fared poorly when applied to a new cohort: PALSUSE, DeFeo, ANCLA, RISK-E, EndoSCORE, MELD-XI, COSTA, and SHARPEN. DeFeo score demonstrated the largest discrepancy with initial AUC of 0.88, compared to 0.58 when applied to different cohorts. The inflammatory response in IE has been well documented and CRP has been found to be an independent predictor for worse outcomes. There is ongoing investigation on alternate inflammatory biomarkers which may assist in IE management. Of the scores identified in this review, only three have included a biomarker as a predictor.

**Conclusion:**

Despite the variety of available scores, their development has been limited by small sample size, retrospective collection of data and short-term outcomes, with lack of external validation, limiting their transportability. Future population studies and large comprehensive registries are required to address this unmet clinical need.

## Introduction

Infective endocarditis (IE) is a rare, highly morbid condition, affecting 6.8 patients per 100,000 per year in the United Kingdom (UK) (1), with an in-hospital mortality rate of 17.1% (2). In the 2019 EURO-ENDO registry data, almost 70% of patients had a theoretical indication for surgery with 51% undergoing surgical intervention (2). The aim of surgery in this group of patients is removal of the vegetation/infection source and repair/replacement of the valve involved to restore function (3). Despite the advances in diagnostic testing, antibiotic therapy and surgical techniques, the incidence and mortality of IE has remained largely the same over the past 30 years (4).

By virtue of its pathophysiology, care of IE patients requires a multi-specialty approach, involving cardiologist, microbiologist, surgeon, intensivist and imaging specialist at the very least. The 2015 ESC (European Society of Cardiology) Guidelines for the management of IE emphasize this approach in the form of an “endocarditis team,” recommending prognostic assessment based on clinical, microbiological and echocardiographic data (5). However, according to both literature and clinical practice, there is no prognostic tool available that encompasses these three levels of information, collected within 48–72 h from admission.

The need for a modern, comprehensive and widely applicable predictive score for risk stratification of this diverse patient group is essential for decision-making within the Endocarditis Team. A validated risk score encompassing the triad of clinical, microbiological and imaging characteristics (5) would be a useful tool to help define prognosis and management.

Published risk scores have been limited to small, very specific patient groups spanning a long period of time. In addition, many of the scores have been developed specifically for surgical cohorts, treated in tertiary centers, excluding patients with implantable cardiac devices or prosthetic valves.

This systematic review aims to synthesize the data on predictive models reported in the literature intended to guide management decisions during the acute care of adults with IE and assess their reported performance in the clinical setting. This data will highlight areas for development and improved data analysis for IE patients, as well as provide a framework for ongoing research in the identification of a comprehensive predictive score.

## Materials and methods

Standard methodology for systematic review as per Preferred Reporting Items for Systematic Reviews and Meta-Analyses (PRISMA) guidelines which can be accessed on *prisma-statement.org*.

Search for existing literature in Medline (*via* PubMED) and EMBASE (*via* OVID) databases with the keywords: < “infective endocarditis” AND “risk score” > from inception until May 2021. Records were independently assessed by two separate reviewers and cross-referenced with the senior reviewer in order to reach concordance. Reference lists from relevant studies were also analyzed for suitable research titles.

Studies included involved scoring systems intended to guide treatment during the acute care of adults with IE, such as the need of surgical intervention. Publications which included risk score analysis and assessment were included. The area under the receiver operating characteristic curve (AUC/ROC) for each risk score with details of patient cohorts and their corresponding data were extracted for each paper. AUC compares the sensitivity (true positives) with the specificity (false positives), thus assessing performance and determining accuracy of the multi-factor risk scores (6). Studies describing development only, development and validation and validation only were all included in the review. Studies comparing different scores when applied to new populations were included. Studies reporting non-validated risk scores were also included.

Studies of pediatric or congenital populations, analysis of timing of surgery for IE, assessment of dental practice, case reports and literature reviews were excluded from the review. Studies of scoring systems designed to aid the diagnosis, investigation or prevention of IE were excluded. Research that was only represented by a conference abstract were excluded due to lack of detail for comparison purposes.

Data were extracted according to a structured protocol and included patient demographics, clinical covariates, microbiological results and imaging criteria. Reported outcomes were documented, including mortality and morbidity numerators. Details of risk score assessment, by means of AUC/ROC analysis, were collected and used to qualitatively compare score performance, including sensitivity and specificity where reported.

The scores extracted were individually assessed for risk of bias and applicability to our review using the Prediction model Risk Of Bias Assessment Tool (PROBAST) (7).

Confirmed IE was defined by modified Duke’s Criteria as described by Li et al. (8). The definitions of variables included were the same as those described for the EuroSCORE II model and are elaborated in the tabulated results. Active IE was defined as patients undergoing antibiotic therapy at the time of analysis. Any instance where the definition varied from the above has been described.

## Results

A total of 33 studies were included in the qualitative synthesis based on the inclusion criteria (PRISMA Diagram 1). There were a total of 20 relevant scores, with 14/20 being derived from and created specifically for IE populations: Table 1 (3, 9–20). Non-specific scores are tabulated in Table 2 (3, 9–12, 14, 21–27) and include EuroSCORE I and II, Society of Thoracic Surgery (STS) risk score, Ontario province risk (OPR) score, Charlson Co-Morbidity Index and Sequential Organ Failure Assessment (SOFA) scores. STS for IE score was considered with non-specific scores since although IE patients were considered, no IE specific characteristics (such as intra-cardiac abscess) were evaluated.

**TABLE 1:** Comparison of Risk Scores and their Performance–Risk Scores made for IE populations.

SCORE	First proposed	Time Scale	Number of variables	Score designed to predict	Study type ^Ref^	Patients (*n*)^Ref^	Populations considered^Ref^	AGE^Ref^	AUC^Ref^	External validation
**PALSUSE** (9)	2014	2008–2010	7	In-hospital mortality	**Prospective** (3, 9, 12), Retrospective (11, 28), Observational retrospective (23, 24)	361 (3) **437** (9) 138 (11) 671 (12) 324 (28) 107 (21) 192 (22) 180 (23, 24)	**Surgical patients** with definite IE as defined by modified Duke criteria: (11, 21, 28) **active IE** (9) left-sided active IE (12, 23) native and prosthetic valve ACTIVE IE (24) including implantable cardiac devices (3) MEDICAL patients with definite IE (Mod Duke’s) not considered for surgery. Incl all valves and devices (22)	59.1 ± 15.4 (3) **61.4 ± 16.4** (9) 60 ± 8.5 (11) 61 ± 14 (12) 61.8 ± 14.6 (28) 58.1 ± 14.5 (21) 65.2 ± 15.2 (22) 63.4 ± 13.8 (23) 63.2 ± 1 (24)	0.684 (0.633–0.731) (3) **0.84 (CI 0.79–0.88)** (9) 0.694 (CI 0.610–0.770) (11) 0.64 (CI 0.58–0.68) (12) 0.703 (CI 0.650–0.752) (28) 0.68 (CI 0.57–0.79) (21) 0.695 (CI 0.598–0.792) (22) 0.73 (CI 0.66–0.79) (23) 0.73 (CI 0.66–0.79) (24)	No
**De Feo Score** (10)	2012	1980–2009	6	Post-operative mortality (in-hospital/30-day)	**Prospective** (3, 10) Retrospective (11, 22, 25, 28) Observational retrospective (23, 24)	361 (3) **440** (10) 138 (11) 324 (28) 192 (22) 180 (23, 24) 146 (25)	**Surgical patients** with definite IE as defined by modified Duke criteria: (11, 28) **native valve IE only** (10) left-sided active IE (23) native and prosthetic valve ACTIVE IE (24, 25) including implantable cardiac devices (3) MEDICAL patients with definite IE (Mod Duke’s) not considered for surgery. Incl all valves and devices (22)	59.1 ± 15.4 (3) **49 ± 16** (10) 60 ± 8.5 (11) 61.8 ± 14.6 (28) 65.2 ± 15.2 (22) 63.4 ± 13.8 (23) 63.2 ± 1 (24) 48.8 ± 16.0 (25)	0.722 (CI 0.654–0.790) (3) **0.88 (CI 0.82–0.93)** (10) 0.695 (CI 0.611–0.771) (11) 0.615 (CI 0.559–0.668) (28) 0.584 (CI 0.489–0.680) (22) 0.68 (CI 0.58–0.76) (23) 0.68 (CI 0.58–0.76) (24) 0.744 (CI 0.590–0.899) (25)	No
**ANCLA score** (11)	2017	2000–2015	5	In-hospital mortality	**Retrospective** (11, 28)	**138** (11) 324 (28)	**Surgical patients with definite IE as** **defined by modified Duke criteria** (11, 28)	**60 ± 8.5** (11) 61.8 ± 14.6 (28)	**ANCLA pre-op** **0.828 (CI 0.754–0.887)** **ANCLA combined** **0.823 (CI 0.749–0.883)** (11) 0.842 (CI 0.798–0.880) (28)	No
**Risk-E** **Endocarditis** **Score** (12)	2017	1996–2014	8	In-hospital mortality	**Prospective** (12) Retrospective (21, 28) Observational retrospective (23)	**671** (12) 324 (28) 107 (21) 180 (23)	**Surgical patients** with definite IE as defined by modified Duke criteria: (21, 28) **left-sided active IE** (12, 23)	**61 ± 14** (12) 61.8 ± 14.6 (28) 58.1 ± 14.5 (21) 63.4 ± 13.8 (23)	**0.82 (CI 0.75–0.88)** (12) **0.76 (CI 0.64–0.88) *n* = 204,** **ext validation sample** (12) 0.669 (CI 0.615–0.720) (28) 0.71 (CI 0.60–0.81) (21) 0.76 (CI 0.78–0.82) (23)	**Yes** (12)
**EndoSCORE** (3)	2017	2000–2015	9	30-day mortality	**Retrospective** (13, 21, 22, 28)	**2,715** (13) 324 (28) 107 (21) 192 (22)	**Surgical patients** with definite IE as defined by modified Duke criteria: (21, 28) **native and prosthetic valves** (13) MEDICAL patients with definite IE (Mod Duke’s) not considered for surgery. Incl all valves and devices (22)	**59.6 ± 15.1** (13) 61.8 ± 14.6 (28) 58.1 ± 14.5 (21) 65.2 ± 15.2 (22)	**0.851 (CI 0.845–0.858)** (13) 0.663 (CI 0.609–0.715) (28) 0.76 (CI 0.66–0.86) (21) 0.724 (CI 0.634–0.814) (22)	No
**APORTEI** **score** (14)	2020	2008–2018	11	In-hospital/30-day mortality	**Prospective registry** (14) Prospectiv (26)	**1,338** (14) 111 (26)	**Surgical patients** with definite IE as defined by modified Duke criteria (26) **native and prosthetic valve ACTIVE** **IE** (14)	**63.6 ± 13.1** (14) 58.9 ± 13.7 (26)	**0.75 (CI 0.72–0.77)** (14) 0.88 (CI 0.83–0.93) (26)	**Yes** (14)
**AEPEI** **Score I** (3)	2017	2000–2015	5	In-hospital mortality	**Prospective** (3) Retrospective (21, 22, 28)	**361** (3) 324 (28) 107 (21) 192 (22)	**Surgical patients** with definite IE as defined by modified Duke criteria: (21, 28) **including implantable cardiac devices** (3) MEDICAL patients with definite IE (Mod Duke’s) not considered for surgery. Incl all valves and devices (22)	**59.1 ± 15.4** (3) 61.8 ± 14.6 (28) 58.1 ± 14.5 (21) 65.2 ± 15.2 (22)	**0.780 (CI 0.734–0.822)** (3) 0.787 (CI 0.738–0.830) (28) 0.65 (CI 0.53–0.77) (21) 0.654 (CI 0.552–0.756) (22)	**Yes** (3)
**AEPEI** **Score II** **(alternate** **model)** (3)	2017	2000–2015	3	In-hospital mortality	**Prospective** (3) Retrospective (22, 28)	**361** (3) 324 (28) 192 (22)	**Surgical patients** with definite IE as defined by modified Duke criteria: (28) **including implantable cardiac devices** (3) MEDICAL patients with definite IE (Mod Duke’s) not considered for surgery. Incl all valves and devices (22)	**59.1 ± 15.4** (3) 61.8 ± 14.6 (28) 65.2 ± 15.2 (22)	**0.774 (CI 0.727–0.816)** (3) 0.771 (CI 0.722–0.816) (28) 0.633 (CI 0.527–0.739) (22)	**Yes** (3)
**COSTA** (15)	2007	1988–1999	6	Risk of death	**Retrospective** (15), Observational retrospective (24)	**186** (15), 180 (24)	**Medical and Surgical Patients** (15) Surgical patients with definite IE as defined by modified Duke criteria: native and prosthetic valve ACTIVE IE (24)	**33.9** (15) 63.2 ± 1 (24)	**0.872** (15) 0.65 (CI 0.57–0.72) (24)	No
**SHARPEN** (16)	2015	2001–2011	7	In-hospital mortality	**Retrospective** (16)	**233** (16)	**Medical and Surgical Patients** (16)	**50 ± 19** (16)	**0.86 (CI 0.80–0.91)** (16)	No
**Simplified** **Risk Score** **(ICE)** (17)	2016	2000–2006	14	6-month mortality	**Prospective** (17, 19) Retrospective (22)	**4,049** (17) 858 (19) 192 (22)	**Medical and Surgical Patients** (19) **prosthetic valve excluded** **(54.67% surgical)** (17) MEDICAL patients with definite IE (Mod Duke’s) not considered for surgery. Incl all valves and devices (22)	**45–72** (17) 45 ± 15 (19) 65.2 ± 15.2 (22)	**Harrell’s C statistic** **0.715 (CI 0.62–0.89)** (17) 0.771 (19)–long-term mortality, 0.816 (19)–in-hospital death 0.682 for validation model 0.706 (CI 0.617–0.798) (22)	**Yes** (17)
**LOPEZ** (18)	2011	1996–2003	3	In-hospital mortality or urgent surgery	**Retrospective** (18)	Ext Validation 264 (18)	**Surgical patients** with definite IE as defined by modified Duke criteria: **left-sided active IE** (18)	**61 ± 16** (18)	**0.67** **Sensitivity 79%, Specificity** **57%** (18)	**Yes** (18)
**Modified** **MELD-XI** (19)	2018	2009–2015	5	In-hospital/Long-term mortality	**Prospective** (19)	858 (19)	**Medical and Surgical Patients** (19)	**45 ± 15** (19)	**0.823** (19) **in-hospital mortality** **0.730 (CI 0.658–0.803)** (19) **long-term mortality**	No
**Cystatin C** (20)	2012	1999–2005	4	5-year mortality	**Retrospective** (20)	125 (20)	**Medical and Surgical Patients,** **including prosthetic valves and** **cardiac devices** (20)	**62.7 ± 16.9** (20)	**0.74 (CI 0.70–0.87)** (20)	No

The results for the IE specific scores (i.e., the scores created for IE populations) which are delineated in bold/underline denote the studies. In which the risk score in question was first proposed, with data in bold referring to the derivation cohort. All other data (not in bold) for each score, include studies where the score in question has been used in comparison to other scores. All scores which have been validated (either described in the same paper or in a separate paper) have been identified in the last column, with the paper reference indicated accordingly in the last column.

**TABLE 2:** Comparison of Risk Scores and their Performance–Risk Scores not specific for IE populations.

SCORE	Number of variables	Score designed to predict	Study type^Ref^	Patients (*n*)^Ref^	Populations considered^Ref^	AGE^Ref^	AUC^Ref^
**OPR–Ontario province risk**	6	Mortality, prolonged ICU stay (>6 days), prolonged post-op stay (>17 days)	Prospective (3), Retrospective (11, 22)	361 (3), 138 (11), 192 (22)	Surgical patients with definite IE as defined by modified Duke criteria: (11) including implantable cardiac devices (3) MEDICAL patients with definite IE (Mod Duke’s) not considered for surgery. Incl all valves and devices (22)	59.1 ± 15.4 (3) 60 ± 8.5 (11) 65.2 ± 15.2 (22)	0.698 (CI 0.647–0.745) (3) 0.637 (CI 0.661–0.717) (11) 0.669 (CI 0.573–0.765) (22)
**The Society of Thoracic Surgery (STS) risk score** **OR STS for IE score**	12	Post-operative mortality and morbidity	Prospective (3, 12), Retrospective (11, 21, 22, 25, 28), Observational retrospective (23, 24)	361 (3) 138 (11) 671 (12) 324 (28) 107 (21) 192 (22) 180 (23, 24) 146 (25)	Surgical patients with definite IE as defined by modified Duke criteria: (11, 21, 28) left-sided active IE (12, 23) native and prosthetic valve ACTIVE IE (24, 25) including implantable cardiac devices (3) MEDICAL patients with definite IE (Mod Duke’s) not considered for surgery. Incl all valves and devices (22)	59.1 ± 15.4 (3) 60 ± 8.5 (11) 61 ± 14 (12) 61.8 ± 14.6 (28) 58.1 ± 14.5 (21) 65.2 ± 15.2 (22) 63.4 ± 13.8 (23) 63.2 ± 1 (24) 48.8 ± 16.0 (25)	0.709 (CI 0.659–0.756) (3) 0.540 (CI 0.453–0.625) (11) 0.74 (CI 0.68–0.79) (12) 0.742 (CI 0.691–0.789) (28) 0.67 (CI 0.56–0.79) (21) 0.757 (CI 0.676–0.837) (22) 0.76 (CI 0.68–0.82) (23) 0.76 (CI 0.68–0.82) (24) 0.699 (CI 0.534–0.865) (25)
**EUROSCORE II**	18	In-hospital mortality	Prospective (3, 12, 26), Retrospective (11, 20–22, 25), Observational retrospective (23, 24)	361 (3) 138 (11) 671 (12) 465 (20) 107 (21) 192 (22) 180 (24) 146 (25) 111 (26)	Surgical patients with definite IE as defined by modified Duke criteria: (11, 21, 26, 28) left-sided active IE (12) native and prosthetic valve ACTIVE IE (24, 25) including implantable cardiac devices (3) Surgical patients diagnosed with IE depending on blood culture and intra-op findings (20) MEDICAL patients with definite IE (Mod Duke’s) not considered for surgery. Incl all valves and devices (22)	59.1 ± 15.4 (3) 60 ± 8.5 (11) 61 ± 14 (12) 50 ± 16.9 (20) 58.1 ± 14.5 (21) 65.2 ± 15.2 (22) 63.2 ± 1 (24) 48.8 ± 16.0 (25) 58.9 ± 13.7 (26)	0.751 (CI 0.704–0.795) (3) 0.733 (CI 0.683–0.831) (11) 0.76 (CI 0.70–0.82) (12) 0.816 (20) 0.69 (CI 0.58–0.8) (21) 0.773 (CI 0.704–0.843) (22) 0.74 (CI 0.66–0.82) (24) 0.656 (CI 0.466–0.846) (25) 0.74 (CI 0.69–0.79) (26)
**EUROScore I**	17	In-hospital mortality	Prospective (9, 10, 12, 26), Prospective registry (14), Retrospective (11, 21, 22, 25), Observational retrospective (23, 24)	437 (9) 440 (10) 138 (11) 671 (12) 1,338 (14) 107 (21) 192 (22) 180 (23, 24) 146 (25) 111 (26)	Surgical patients with definite IE as defined by modified Duke criteria: (11, 21, 26) active IE (9) left-sided active IE (12, 18, 23) native valve IE only (10) native and prosthetic valve ACTIVE IE (14, 24, 25) MEDICAL patients with definite IE (Mod Duke’s) not considered for surgery. Incl all valves and devices (22)	61.4 ± 16.4 (9) 49 ± 16 (10) 60 ± 8.5 (11) 61 ± 14 (12) 63.6 ± 13.1 (14) 58.1 ± 14.5 (21) 65.2 ± 15.2 (22) 63.2 ± 1 (24) 48.8 ± 16.0 (25) 58.9 ± 13.7 (26)	0.73 (CI 0.70–0.77) (9) 0.84 (CI 0.77–0.91) (10) Additive 0.733 (CI 0.651–0.805) Logistic 0.658 (CI 0.572–0.736) (11) 0.76 (CI 0.71–0.82) (12) 0.72 (CI 0.69–0.75) (14) 0.77 (CI 0.66–0.86) (21) 0.777 (CI 0.710–0.844) (22) 0.74 (CI 0.66–0.82) (24) Additive 0.653 (CI 0.487–0.819) Logistic 0.645 (CI 0.487–0.803) (25) 0.77 (CI 0.72–0.82) (26)
**SOFA–Sequential Organ Failure Assessment**	8	In-hospital/intensive care mortality	Retrospective (27)	66 (27)	Surgical patients with definite IE as defined by modified Duke criteria (27)	70 (19–88) (27)	0.915 (CI 0.845–0.986) (27)
**Charlson Co-Morbidity Index**	14	10-year mortality	Retrospective (27)	66 (27)	Surgical patients with definite IE as defined by modified Duke criteria (27)	70 (19–88) (27)	0.788 (CI 0.655–0.922) (27)

AEPEI, SHARPEN and Cystatin C scores, although made for IE populations, did not include specific variables pertaining to IE such as: micro-organism, embolic events, degree of myocardial/valve damage, abscess formation and large vegetations, although these were evaluated. Discrimination performance was classified depending on AUC: Excellent with AUC 0.9–1.0, good with AUC 0.8–0.9, fair with AUC 0.7–0.8, poor with AUC 0.6–0.7, and very poor with AUC 0.5–0.6 (28).

The scores assessed in this review and corresponding AUC results from different studies are tabulated in Tables 1, 2 (3, 9–28). Of these scores, only six have been externally validated in a separate cohort (3, 12, 14, 17, 18). EuroSCORE I was most often used for comparison, being analyzed in 11 separate studies with a consistently fair performance. EuroSCORE II was reviewed in 10 studies, STS-IE and PALSUSE score were reviewed in nine studies each. STS-IE had an overall fair discrimination performance across studies, with only 3/9 studies with an AUC < 0.7 (11, 21, 25). The PALSUSE score was frequently included in comparative studies but performed overall poorly with an AUC < 0.7 in five of these comparative studies (3, 11, 12, 21, 22).

### Statistical methods and selection of variables

The majority of studies identified variables for inclusion in the risk score by multivariate logistic regression analysis. The exception was Park et al. in the development of the simplified risk (or ICE) score, where the variables considered were selected *a priori* by an experienced cardiologist (17) before analysis for significance. Martinez-Sellis et al. in the development of the PALSUSE score utilized stepwise logistic regression analysis (9).

**FIGURE 1:**
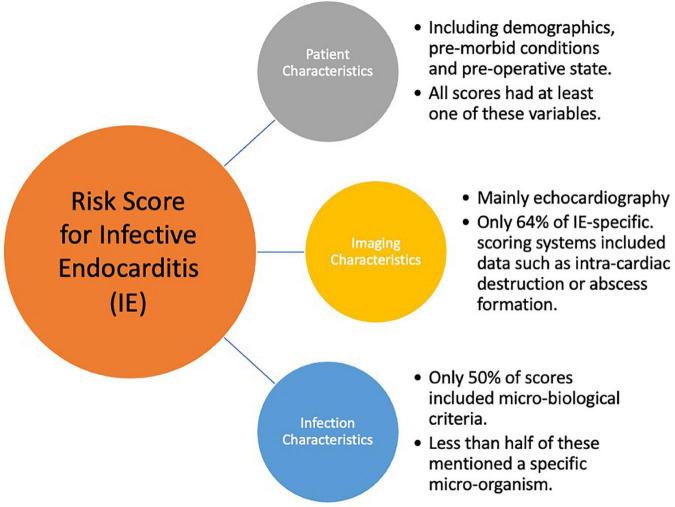
Key components for an Infective Endocarditis Risk Score.

**FIGURE 2:**
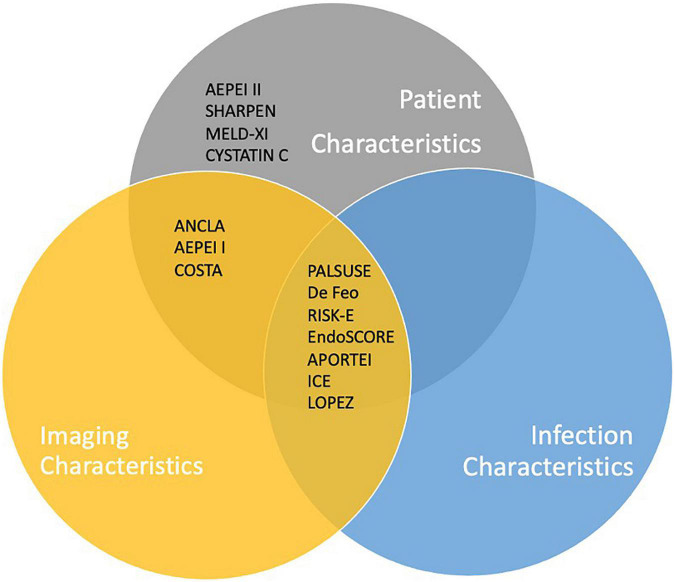
Inclusion of key criteria in IE-specific risk scores currently available.

The number of variables assigned to each score and the variables included have been divided into three broad categories: Clinical variables (patient demographics, co-morbid and acute physiological state); Imaging characteristics (mainly echocardiographic findings); Microorganisms. Figures 1, 2 are graphic representations of the scores within these categories. Variables included for each score are depicted in Supplementary Table 1.

Of the IE-specific scores, 7/14 included microbiology criteria: Four studies considered a positive blood culture and four considered the presence of *Staphylococcus aureus* within their score. ICE score included both, as well as the presence of “Viridans streptococci.” The scores which did not include microorganisms in their model (De Feo, RISK-E, AEPEI I and II, COSTA, SHARPEN, MELD-XI, and CYSTATINC) had access to microorganism data for their patient set; however, the microorganism was not found to be a significant factor in univariate and multivariate analysis and was subsequently excluded from their model.

### Discrimination performance

Good discrimination performance of the following scores PALSUSE, De Feo, ANCLA, RISK-E, EndoSCORE, COSTA, and SHARPEN, were only identified in the studies proposing the score and most often in the derivation cohort. In follow-up, validation and other comparative studies, this result was not replicated. The only score to repeatedly score an AUC > 0.8 was the ANCLA score which was included in only two studies by the same first author.

EuroSCORE I, II, and STS-IE were most frequently used for comparison purposes with a fair performance (AUC 0.7–0.8). Relative difference between the best and worst AUC estimates for each score ranged from 15 to 34%. Many of the scores performed fairly (AUC 0.7–0.8) when compared in other studies. As expected, the performance was below that described in the original derivation cohort studies for these scores (Tables 1, 2).

### Calibration and model performance

Inter-model comparisons were provided for 14 studies: Hosmer-Lemeshow Test Statistic was used in 10 reports, Calibration slope provided in two studies and U statistic in one study. In the majority of calibration studies, the risk scores analyzed were found to be adequately calibrated.

The EuroSCORE II was found to have inaccurate calibration in one study (24) which authors attributed to the lack of specific IE factors in the score, however; in the same study, EuroSCORE I (lacking the same IE-specific factors) had adequate calibration. The Brier score, analyzing the difference between prediction and actual outcome, with a result of 0 being perfect, has been utilized in only one study proposing the EndoSCORE, quoting a Brier score of 0.078 (13).

### Outcome selection bias

Long-term outcome data for IE patients is often unavailable. The majority of studies considered in-hospital mortality or mortality within 30 days as the primary end-point (3, 9–14, 16, 19, 22–25, 28), some interchangeably. Other end-points included 6-month mortality (17), urgent surgery OR in-hospital mortality (18) and long-term mortality of 29 months (19) and 5 years (20).

The different population cohorts assessed are specified in Tables 1, 2. While all studies defined IE using the modified Duke’s criteria, some opted to only include patients with active IE. Only seven studies included medically managed patients (15–17, 19, 20, 22, 27), with others including surgical patients only. ICE, SHARPEN, Modified MELD-XI, Cystatin C, and COSTA scores were developed from cohorts with both medical and surgical patients. The COSTA score performed poorly when later applied to a surgical cohort (24). The ICE score maintained fair discrimination performance when applied to an exclusively medically treated cohort (22). Furthermore, only three papers included patients with implantable cardiac devices (3, 20, 22).

### Overall risk of bias

In the PROBAST assessment (Supplementary Table 2), the majority of studies were found to have high risk of bias in participant choice due to the specific populations considered (e.g., surgical patients only, native valve only etc.). This systematic review aims to encompass scores that incorporate the whole of the IE population if possible. The simplified risk score (ICE) is the score with the least risk of bias; however, it is one of the few scores assessing a long-term outcome of 6 month mortality rather than in-hospital or 30 day mortality, making it difficult to compare with the other scores available. Moreover, it has over double the variables of the other scores, making it less user-friendly.

## Discussion

This systematic review has highlighted important limitations that preclude the transportability of published risk-scores to various IE groups in different healthcare settings and regions. The challenge with risk stratification and accurate prognostication in IE is largely due to the heterogenous patient population affected. The majority of scoring systems identified address the issue of surgical risk, therefore being unable to estimate mortality risk for medically treated populations.

The IE patient is now wholly different from the one 30 years ago. Percutaneous vascular interventions have become more commonplace, as have the number of cardiac implantable devices. IE associated with cardiac devices has been reported in up to 7% of cases (29), coinciding with a rise in staphylococcal infections (4). There is also an increase in prosthetic valve endocarditis. These under-represented groups of patients are often excluded from the outset in the development cohorts for predictive scoring.

The prevalence of intravenous drug users presenting with IE is also on the rise, with cases doubling between 2008 and 2014 (30). They tend to be younger, more acutely unwell patients, with infection caused by gram-positive pathogens (31).

### Clinical impact of IE risk-scoring

Infective endocarditis remains a highly morbid and highly fatal condition, in spite of advances in imaging, improvements in microbiological testing, antibiotic therapy and surgical treatment. Ideally scores should be available within 48–72 h of patient admission into hospital, to guide early management decisions. Lopez et al. only examined variables available within 72 h of admission (18). Possible routes of infection are multiple, with data available at different time-points and not necessarily standardized for all patients. Number and frequency of blood cultures taken may vary, as well as access to trans-thoracic/trans-oesophageal echocardiography (TTE/TOE).

Recent EURO-ENDO registry data showed that for patients in which surgical intervention was found to be necessary, 22.5% died before surgery could be performed (2). This highlights the need for quick and effective decision making which would be significantly easier with a reliable risk tool. Pooling of different IE groups may allow differentiation of risk between the groups within the tool.

Surgical intervention is often carried out as an emergency or urgent procedure after evidence of embolization, heart failure or in the presence of uncontrolled infection (32). This is a complex decision with surgery in the active phase often associated with significant risk. For example, patients with new neurology may experience peri-operative cerebral bleeding with early cardiac surgery intervention. There is variation between studies in the definition of “early surgery” and the results are inconclusive (33). The heart team meeting is essential in making decisions about timing of intervention and while it is beyond the scope of this review, risk-scoring has the potential role in guiding a more accurate selection process toward optimal timing of surgery.

### Validity of risk scores

The ideal risk score should have easily measurable parameters which are comparable across centers (13), clear definitions of predictive parameters and outcomes to ensure widespread use, as well as generalisability to future patients and transportability to other data-sets/patients, determined through a robust validation process (34, 35). Predictors should be easy to collect and the result of cheap and non-invasive testing (36).

Only 6 of the 14 IE-specific scores proposed have undergone formal external validation, limiting their transportability. The absence of externally validated scores has been highlighted multiple times in the literature and analysis of the European IE-Registry (EURO-ENDO) was proposed to achieve this aim (28). Despite the lack of external validation, many scores have been frequently re-assessed in separate studies with different cohorts.

Comparison of the AUC/ROC for the same score between studies allows for understanding of model performance in different IE groups. De Feo score performed poorly in most comparative studies (11, 22–24, 28); however, this score was derived from a small specific cohort of patients with left-side only native valve endocarditis (10), which may explain the inaccurate results when applied to different populations. In addition, it was developed for patients treated from 1980 to 2009. As can be expected, the management of patients in 1980 would have differed significantly from that in 2009, as has the nature of the disease.

EuroSCORE I and II had the least favorable performance in the study by Wang et al. (25), potentially due to the very young average age of their study group (48.8 ± 16 years). There is likely to be an increased significance of the specific IE variables in this young age bracket, which are not included in the EuroSCOREs.

Less than 50% of studies carried out model calibration or performance assessment. Model calibration assesses congruence between model prediction and observed outcome (37). The power of Hosmer-Lemeshow “goodness-of-fit” test increases with sample size and its interpretation in small cohorts such as these, may be inaccurate (38). The use of newer, more advanced methods of performance assessment, such as Brier scoring are known to support risk score use in the clinical setting (37). This should be emphasized going forward, to allow for detailed comparative studies between available scores.

### Prioritization of variables

A fundamental drawback in 7/14 IE specific risk scores is the absence of microbiology from the predictive models. In contrast, the literature demonstrates *Staphylococcus aureus* to be the most common causative microorganism in IE worldwide (39) with strong evidence to suggest its association with worsened morbidity/mortality. ESC guidelines highlight positive blood cultures at 3 days of antimicrobial treatment as an independent risk factor for in-hospital death (5). Investigations vary in different centers and risk scores may standardize this process (e.g., frequency of blood cultures).

Two scores missing microbiology predictors (AEPEI, COSTA) included patients with non-active IE (not undergoing antibiotic treatment at the time of analysis/surgery) at rates of 28.5 and 36%, respectively. The effect of the causative microorganism in patients outside the active phase of IE may be less relevant to outcome and may be the reason for lack of significance in these patient groups.

The PALSUSE score includes EuroSCORE II >10 as a variable. This is a potential confounding factor due to age, gender and urgency of surgery being variables in both PALSUSE and EuroSCORE II, therefore doubling the effect of these variables (9).

Biomarkers feature in only three scores in this review (16, 19, 20), with the most commonly used being C-reactive protein (CRP) of different values. CRP has been found to be an independent predictor for worse outcomes in IE, including an increased risk of embolic events (40), surgical intervention (41), and in-hospital mortality (42). In addition, improvement in CRP was a good predictor of long term outcomes (41).

Furthermore, biomarkers such as sensitive troponin I, interleukin-15 and C-C-chemokine-ligand-4 have been shown in separate studies to predict mortality in IE patients; however, this data is limited to small cohorts (43, 44). The inflammatory response in IE is well documented and is different to other infections (43). Mapping of pro-inflammatory cytokines may be key in risk stratification models to guide early decisions for more aggressive treatment, including surgical intervention.

### The effect of novel diagnostic/treatment on risk-scoring

Developing surgical techniques may have a significant impact on prognosis. Destruction of both the aortic and mitral valves is one of the more challenging presentations of IE; however the “commando” procedure with reconstruction of the aortic-mitral curtain and replacement of both valves has been performed with good results (45). Due to small patient samples for major surgical reconstruction, it is difficult to assess the impact of novel procedures on risk.

Improved patient outcomes have been repeatedly shown for specialized high-volume centers; however, this has not yet been explored for IE patients. Involvement of multiple valves has nonetheless been reflected in some models (13, 14).

Echocardiography is a key tool for prognostication in IE, as reflected in multiple guidelines. The advent of 4D-echocardiography and TOE (pre and intra-operatively) has allowed for detailed understanding of intra-cardiac damage secondary to infection (46). Destructive valve lesions, abscesses and vegetations (with embolization risk) can be identified and are crucial for surgical planning (46).

The use of computed tomography (CT) and F-fluorodeoxyglucose positron emission tomography (PET) has increased particularly for prosthetic or device-related IE; however, regional differences are evident with their use being more common in Western Europe (2). Novel imaging techniques may be incorporated into risk scoring systems for IE and recommendations for use may be found in the ESC guidelines (5).

### Population bias and other limitations in available risk scoring models

Patients with IE undergoing surgery (vis-à-vis most published risk scores) may have a survival advantage as they are already deemed fit for surgery and/or have survived to surgery. There was a particularly high mortality rate reported in the EURO-ENDO registry for patients with indications for surgery who did not undergo surgery (2).

On the flip side, patients may be “too-well” to require surgery due to minimal intra-cardiac destruction and effective response to medical management. Published risk scores fail to capture “antibiotic responders,” especially since they are often managed outside tertiary centers. This effect has been highlighted previously and many risk scores, when tested on medical IE patients NOT considered for surgery, fared quite poorly (22).

Advanced model performance assessment is often missing and the majority of scores have not been externally validated. The limitation of these scores are an extension of the constraints within the studies that propose them. The recurring stumbling blocks include small groups of patients, collected over long time-spans; heterogeneous populations (e.g., left-sided IE, exclusion of cardiac devices, and medical patients) with a focus on tertiary centers, creating a referral bias; single center or regional studies which may not be applicable elsewhere; retrospective analysis (including of prospectively collected data) with certain variables often missing; definition of variables inconsistent across studies and analysis of short-term outcomes with a consistent lack of long-term data.

The lack of long-term data is a significant draw-back, with the majority of the papers reviewed here considering 30 day or in-hospital mortality as the end-point or primary outcome. There is limited data to understand what survival and morbidity, or even quality of life, is like beyond this date. The MDT is still unable to guide patients toward reasonable expectations of what their recovery might involve.

Moreover, it has proved difficult to capture the impact on patient outcomes as a result of delay in diagnosis, time to initiation of treatment and time of referral to specialized care in tertiary centers. The point of referral to an MDT is heterogenous across populations and difficult to assess. This emphasizes the need for a standardized prospective registry encompassing data from the initial clinical presentation to the end of the patient journey and recovery. The implementation of artificial intelligence has not yet been explored in endocarditis patients. This may identify critical negative prognostic signs through imaging and cytokine response, creating personalized risk models.

## Conclusion

In conclusion, despite the multitude of available IE risk-scores, the lack of adequate score validity limits their clinical utility and widespread applicability in this important group of patients. Being a highly morbid condition with a multifactorial pathophysiology and a heterogenous patient population, the accumulation of large sets of real-world data from future coordinated registries including novel biomarkers will produce more robust prediction models. Future registries should also encompass populations with much wider inclusion criteria and more refined classification systems, thus improving patient-specific prognostication. Improved risk scores will have the potential to empower MDTs with an objective stratification tool to guide management in patients with IE, as well as allow for key comparative studies and improved management strategies for IE.

## Data availability statement

The original contributions presented in this study are included in this article/Supplementary material, further inquiries can be directed to the corresponding author.

## Author contributions

All authors listed have made a substantial, direct, and intellectual contribution to the work, and approved it for publication.
