# Supplementary material for: Infective endocarditis: Do we have an effective risk score model? A systematic review
Source: Front Cardiovasc Med. 2023 Feb 20;10:1093363. doi: 10.3389/fcvm.2023.1093363 (PMC9986297; doi:10.3389/fcvm.2023.1093363)
Supplement: Supplementary file 1 [file Table_1.pdf]

Supplementary Table 1: Comparison of Variables utilised in different Risk Scores

	**Risk Scores**	**Number of Variables**	Patient Characteristics															Pre-operative State					Imaging Characteristics						Micro-organisms			Surgical Characteristics			**Definitions and Other Included Variables** *(Variables present in less than 2 of the scores included below)*
			Age	Gender	BMI	Blood Pressure	Renal impairment[1]	NYHA[2]	Diabetes Mellitus	COPD[3]	Thrombocytopaenia[4]	CRP	Arrythmia	Stroke	Active IE	Prosthetic valve	Previous cardiac surgery	Critical state[4]	Septic shock	Cardiogenic shock[6]	Pre-op inotropes/balloon pump	Ventilator support	LVEF	Large intra-cardiac destruction	Peri-valvular involvement	Abscess	Large mobile vegetation	Systolic Pulmonary Artery Pressure (sPAP) >55 or Pulmonary hypertension	Positive blood culture	Staph aureus	Viridans group streptococci	Urgent/Emergent/Salvage	Number of treated valves/ prostheses/ Type of surgery	Surgery of thoracic aorta	
IE-specific SCORE	PALSUSE	7	X	X												X								X						X		X			<u>Age</u> ≥70, <u>Intracardiac destruction</u>: abscesses, fistulae, wall dissection or valve dehiscence<br>Other: EuroSCOREII ≥10
	De Feo Score	6	X				X	X														X			X				X						<u>Ventilator support</u> included intubated patients and NIV pre-operatively, <u>Positive blood culture</u>: Surgery before attainment of a negative blood culture, <u>Peri-valvular involvement</u>: abscess or fistula
	ANCLA score	5						X										X						X										X	<u>Large intracardiac destruction</u>: extensive valve destruction, perivalvular complications or multivalvular involvement.<br>Other: Anaemia: haemoglobin <12 g/dl (female) or <13 g/dl (male)
	Risk-Endocarditis Score (RISK-E)	8	X				X				X					X			X	X					X				X						<u>Renal failure:</u> glomerular filtration rate <60 mL/min/1.73 m2<br><u>Septic shock</u>: acute circulatory failure in sepsis, with persistent systolic pressure <90 mm Hg despite adequate volume resuscitation
	EndoSCORE	9	X	X			X			X									X				X			X			X				X		
	APORTEI score	11	X	X			X	X								X	X			X						X				X		X	X		<u>NYHA</u> >III.<br><u>Urgent surgery</u>: within 24 hours of indication
	AEPEI Score I	5			X		X	X										X										X							BMI >27, <u>Renal impairment:</u> eGFR <50
	AEPEI Score II (alternate model)	3					X	X										X																	<u>Renal impairment:</u> eGFR <50
	COSTA	6	X										X						X	X				X			X								<u>Cardiogenic shock</u>: vasoactive drugs to maintain adequate pressure and output; <u>Septic shock</u>: fever>3days OR persistant positive blood cultures refractory to adequate antibiotics
	SHARPEN	7	X			X	X					X								X															Systolic BP <90mmHg, Heart Failure: not specified, <u>CRP</u> >200mg/L<br>Other: Pneumonia, Non-intravenous drug abusers
	Simplified Risk Score (ICE)	14	X				X							X		X									X		X		X	X	X				<u>Renal impairment</u>: History of dialysis, <u>NYHA</u> III or IV<br>Other: Nosocomial IE, Symptoms >1month before admission, surgical treatment
	LOPEZ	3																		X					X					X					Heart Failure as defined [7]
	Modified MELD-XI	5					X	X				X																							<u>Renal impairment:</u> for calculation of the MELDXI score as follows: 5.11*(ln total bilirubin, mg/dl)+11.76*(ln creatinine, mg/dl)+9.44.<br><u>NYHA</u> >III; <u>CRP</u> >9.5mg/L<br>Other: Bilirubin, Non-surgical Treatment
	CystatinC	4	X																																<u>Age</u> ≥70, <u>CysC</u> >1.2 mg/l, <u>NT-ProBNP</u> >2000 ng/l, <u>presence of any grade of MI</u>

Supplementary Table 1: Comparison of Variables utilised in different Risk Scores

-Specific Risk Scores	OPR – Ontario province risk	**6**	X	X													X						X									X	X		
	SOFA	**8**					X				X										X	X													Renal impairment: Creat >1.2mg/dL<br>Other: Fi02>21%, Pa02, Bilirubin >1.2, HR >100,
	Charlson Co-Morbidity Index	**14**	X				X		X	X				X																					Renal impairment: moderate-severe<br>Other: MI, CHF, Dementia, connective tissue disease, Peptic ulcer disease, Liver disease, Leukaemia, Lymphoma, AIDS
	The Society of Thoracic Surgery (STS) risk score for IE	**12**				X	X		X	X			X		X		X			X	X											X	X		Blood Pressure: Hypertension<br>Emergency/salvage status or cardiogenic are considered as a single variable; urgent cases without cardiogenic shock are considered as a separate variable. Insulin-dependent and non-insulin dependent diabetes mellitus are considered as separate variables.
	EUROSCORE I	**17**	X	X			X			X					X		X	X					X					X				X		X	Other: extracardiac arteriopathy, neurological dysfunction, unstable angina, Recent MI, other than isolated CABG, post infarct septal rupture
	EUROSCORE II	**18**	X	X			X	X	X	X					X		X	X					X					X				X		X	Other: extracardiac arteriopathy, poor mobility, CCS IV, unstable angina, Recent MI, Weight of intervention, post infarct septal rupture DM on insulin.

Unless indicated otherwise, the definitions for the variables above are as follows:
[1] Serum Creatinine >2mg/dL
[2] New York Heart Association (NYHA) Classification Score of IV unless indicated otherwise
[3] Thrombocytopaenia: platelet count <150 000/mL
[4] Chronic Obstructive Pulmonary Disease (COPD): long-term use of bronchodilators or steroids for lung disease
[5] Critical State: ventricular tachycardia or ventricular fibrillation or aborted sudden death, pre0operative cardiac massage, pre-operative ventilation before anaesthetic room, preoperative inotropes or IABP, preoperative acute renal failure (anuria or oliguria <10ml/hr)
[6] Cardiogenic Shock: systolic blood pressure <90 mm Hg and tissue hypoperfusion due to myocardial dysfunction, despite adequate resuscitation, and accompanied by low cardiac index and high pulmonary wedge pressure.
[7] The Task Force on Heart Failure of the European Society of Cardiology Guidelines for the diagnosis of heart failure 1995 [35].
