# Supplementary material for: Infective endocarditis: Do we have an effective risk score model? A systematic review
Source: Front Cardiovasc Med. 2023 Feb 20;10:1093363. doi: 10.3389/fcvm.2023.1093363 (PMC9986297; doi:10.3389/fcvm.2023.1093363)
Supplement: Supplementary file 2 [file Table_2.pdf]

Supplementary Table 2: Prediction model Risk Of Bias Assessment Tool (PROBAST)

STUDY	SCORE	STUDY TYPE	Risk of Bias (ROB)				Applicability			Overall	
			**Participants**	**Predictors**	**Outcome**	**Analysis**	**Participants**	**Predictors**	**Outcome**	**ROB**	**Applicability**
Martinez-Sellís M, Muínoz P et al.[9]	PALSUSE	Development only	-	+	+	-	?	+	+	-	+
			Participants undergoing medical treatment and later those with implantable devices excluded; Use of univariate analysis, Exclusion of subgroups of patients, No information RE handling of missing data, No internal validation								
De Feo M, Cotrufo M et al.[10]	DE FEO	Development only	-	+	+	-	?	+	+	-	+
			Left sided native valve active surgically managed patients only, <20 events per variable (EPV) for development cohort (40 patients), No information RE handling of missing data, No internal validation								
Gatti G, Benussi B et al.[11]	ANCLA	Development only	-	+	+	-	?	+	+	-	+
			Surgical patients only. <20 EPV for development cohort (28 patients), No information RE handling of missing data, No internal validation								
Olmos C, Vilacosta I et al.[12]	RISK-E	Development and validation	-	?	+	-	?	+	+	-	+
			Surgical patients with left-sided IE only. Duke criteria used up to 2002 and modified Duke criteria thereafter. Only 68 patients with the outcome in the validation sample – PROBAST recommends >100.								
Di Mauro M, Dato GMA et al.[13]	EndoSCORE	Development only	-	+	+	+	?	+	+	-	+
			Surgical patients; No information RE handling of missing data								
Varela Barca L, Fernandez-Felix BM et al.[14]	APORTEI	Validation only	-	+	+	+	?	+	+	-	+
			Surgical patients only, 342 patients with the outcome (>100 as per guidelines for a validation study); No information RE handling of missing data								
Gatti G, Perrotti A et al.[3]	AEPEI I & II	Development only	-	+	+	-	?	+	+	-	+
			Surgical patients only. <20 EPV for development cohort (56 in-hospital death); No information RE handling of missing data								
Costa MAC, Wollman DR et al.[15]	COSTA	Development only	+	+	+	-	+	+	+	-	+
			<20 EPV for development cohort (49 in-hospital death); No information RE handling of missing data								
Chee OZ, Tan YQB et al.[16]	SHARPEN	Development only	+	+	+	-	+	+	+	-	+
			<20 EPV for development cohort (54 in-hospital death); No information RE handling of missing data								
Park LP, Chu VH et al.[17]	Simplified Risk Score (ICE)	Development and validation	+	+	+	?	+	?	+	+	+
			Prosthetic valves excluded; selection of predictors made a priori by an experienced cardiologist								
Lopez J, Fernandez-Hidalgo N et al. [18]	LOPEZ	Development and validation	-	?	+	+	?	+	?	-	?
			Surgical patients with left-sided active IE only; Duke criteria used up to 2002 and modified Duke criteria thereafter; Sufficient EPVs for the development study; 132 and 122 patients with the outcome for internal and external validation respectively (>100 as per guidelines for a validation study). Outcome included in-hospital mortality AND/OR urgent surgery								
He PC, Wei XB et al.[19]	Modified MELD-XI	Development only	+	+	+	-	+	+	+	-	+
			<20 EPV for development cohort (70 in-hospital death)								
Bjurman C, Snygg-MartinU et al.	CystatinC	Development only	+	-	+	-	+	-	?	-	+
			Grade of myocardial infarction as predictor not clearly defined, <20 EPV for development cohort (42 death at 5 years)								
EPV: Events per variable; + indicates low ROB/low concern regarding applicability; - indicates high ROB/high concern regarding applicability; ? indicates unclear ROB/unclear concern regarding applicability											
